# Immediate induction versus expectant management in primiparas presenting with decreased fetal movements at 39 weeks

**DOI:** 10.1007/s00404-026-08303-8

**Published:** 2026-01-06

**Authors:** Raneen Abu Shqara, Kylie Ella Marcovich, Yara Nakhleh Francis, Yara Bishara, Nadir Ganem, Lior Lowenstein, Maya Frank Wolf

**Affiliations:** 1 Raya Strauss Wing of Obstetrics and Gynecology, Galilee Medical Center, Nahariya, Israel; 2 Azrieli Faculty of Medicine, Bar Ilan University, Safed, Israel

**Keywords:** Decreased fetal movements, Induction of labor, Expectant management, Primiparous patients, Maternal outcomes, Neonatal outcomes

## Abstract

**Objective:**

To evaluate maternal and neonatal outcomes in primiparous patients presenting with decreased fetal movements (DFM), comparing immediate induction of labor with expectant management.

**Study design:**

This retrospective cohort study included nulliparous patients with singleton pregnancies who presented to our obstetric triage unit between 39 + 0 and 39 + 6 weeks of gestation with a subjective complaint of DFM, a reassuring fetal assessment, and a normal biophysical profile. Patients were offered labor induction. Those who agreed formed the induction group, while those who declined and were discharged home comprised the control group. Maternal and neonatal outcomes were compared.

**Results:**

A total of 413 patients were included: 282 in the induction group and 131 in the expectant management group. Gestational age at delivery was lower in the induction group (39.4 ± 0.3 vs. 40.1 ± 0.3 weeks, p < 0.001). No significant differences were observed in birthweight, cesarean delivery rates, or neonatal intensive care unit (NICU) admission. The induction group had significantly fewer neonates with cord pH < 7.15 (2.5% vs. 6.9%, p = 0.034), and a shorter duration of neonatal hospitalization (2.0 ± 0.2 vs. 2.3 ± 0.5 days, p = 0.034); however, 5-min Apgar scores were similar between the groups. Total maternal hospitalization duration was significantly longer in the induction group (3.6 ± 0.6 vs. 2.2 ± 0.5 days, p < 0.001), though postpartum stay was slightly shorter (2.0 ± 0.2 vs. 2.2 ± 0.1 days, p < 0.001).

**Conclusion:**

Among primiparous patients presenting with DFM between 39 + 0 and 39 + 6 weeks, labor induction was associated with earlier delivery and improved umbilical cord pH without increasing maternal or neonatal complications.

## What this study adds to clinical practice

**Table:** 

This study provides new evidence to guide the management of primiparous patients presenting at 39 weeks with decreased fetal movements (DFM) and a reassuring fetal assessment. Immediate induction of labor was associated with earlier delivery and improved umbilical cord pH values, without increasing rates of cesarean delivery, maternal complications, or neonatal morbidity. These findings support offering induction as a safe option in this clinical scenario while recognizing that expectant management with close surveillance also remains appropriate. This evidence may assist clinicians in counseling patients and individualizing care plans when DFM occurs at term.	

## Introduction

Decreased fetal movements (DFM) in late pregnancy are linked to higher risks of adverse outcomes, including stillbirth, neonatal morbidity, and maternal anxiety [1–3]. The diagnosis of DFM remains challenging owing to the absence of universally accepted diagnostic criteria. Among the various assessment strategies, the 'count-to-ten' fetal movement counting method—whereby the pregnant individual records the time taken to perceive ten discrete movements—is the most widely adopted approach in clinical practice and is endorsed by many obstetric care providers [4, 5]. The rate of DFM at term is approximately 5–6% of pregnancies, based on large cohort studies of women presenting for evaluation of reduced fetal movements after 36 weeks’ gestation, with the majority of cases occurring after 39 weeks [6]. Pregnancies in which patients report DFM have been linked to higher rates of adverse perinatal outcomes, including an increased risk of stillbirth, low Apgar scores, neonatal intensive care unit (NICU) admission, and small-for-gestational-age neonates [7–10]. Despite these associations, data on long-term outcomes remain limited. In a large population-based cohort of over 240,000 deliveries, maternal reports of DFM were not associated with adverse perinatal outcomes or perinatal mortality, but were independently associated with a higher risk of long-term neurological morbidity in the offspring (adjusted HR 1.54, 95% CI 1.0–2.37), particularly movement disorders [11]. Management of primiparous patients presenting with DFM at term, particularly at 39 weeks’ gestation, remains a matter of ongoing debate. Immediate induction of labor and expectant management are the two most commonly considered approaches, each with potential benefits and risks for both mother and fetus [12]. Immediate induction aims to potentially reduce perinatal morbidity by expediting delivery [13]. However, clinical decisions are often individualized and continue to be guided largely by expert opinion and institutional protocols due to the lack of definitive consensus from high-quality evidence [14]. Internationally, the management of DFM varies substantially, especially in late-term pregnancies. In the United Kingdom and several European settings, national initiatives have promoted increased maternal awareness and standardized evaluation pathways for DFM [15, 16]. The Awareness of Fetal Movements and Fetal Mortality (AFFIRM) trial [16]—a large randomized study of more than 400,000 pregnancies—implemented a structured care package that improved the timeliness and consistency of assessment but did not reduce stillbirth rates and was associated with higher induction and cesarean rates, raising concerns about potential overtreatment. Similar variability exists in Australia, where prompt evaluation is recommended but protocols differ across institutions, and increased surveillance has not consistently translated into improved perinatal outcomes [15, 17]. In contrast, guidelines in the United States, including those from the American College of Obstetricians and Gynecologists (ACOG) [18], recommend a single antenatal assessment for an isolated episode of DFM at term when the initial evaluation is reassuring. This international heterogeneity underscores the absence of consensus on optimal management of DFM at 39 weeks and highlights the clinical uncertainty surrounding decisions regarding induction versus expectant management. The ARRIVE trial [13] demonstrated that elective induction at 39 weeks in low-risk nulliparous patients was associated with lower rates of cesarean delivery and hypertensive complications compared with expectant management. However, this trial did not evaluate patients presenting with DFM. Accordingly, high-quality data specifically addressing optimal management of DFM at 39 weeks are lacking. This study aims to compare outcomes of immediate induction versus expectant management in primiparous patients presenting with DFM at 39 weeks, addressing a clinically important question with significant implications for both maternal and fetal health.

## Materials and methods

### Study design

This was a retrospective cohort study conducted at a single tertiary university-affiliated medical center. The study included nulliparous patients with singleton pregnancies who presented to the obstetric triage unit between 39 + 0 and 39 + 6 weeks of gestation (March 2020–March 2023) with a subjective complaint of DFM. Eligibility criteria required a reassuring fetal assessment, and a normal biophysical profile. Exclusion criteria included high-risk pregnancies—such as fetal growth restriction, major fetal anomalies, intrauterine fetal demise, multiple gestation, or maternal conditions requiring individualized high-risk management, including contraindications to vaginal delivery. Pregnancies with well-controlled gestational diabetes or stable chronic hypertension without end-organ involvement were not excluded. Data were extracted from the hospital’s electronic medical records system. The institutional review board of Galilee Medical Center approved the study (0257–24-NHR).

### Exposure

In accordance with departmental protocol, patients presenting with DFM at term were offered immediate induction of labor. Those who agreed formed the induction group, while those who declined and were discharged home comprised the expectant management group. Induction was guided by the Bishop score: patients with a score < 8 underwent cervical ripening with a balloon catheter or dinoprostone, while those with a score ≥ 8 received oxytocin infusion.

### Outcomes

Maternal demographic and obstetric characteristics, intrapartum variables, and maternal and neonatal outcomes were collected from electronic medical records. The primary maternal outcome was cesarean delivery rate. Secondary outcomes included gestational age at delivery, duration of labor, indication for cesarean delivery (non-reassuring fetal heart rate or lack of progress), duration of rupture of membranes, fever and clinical chorioamnionitis, and length of hospitalization (total and postpartum). Neonatal outcomes included birthweight, Apgar score at 5 min, umbilical cord pH < 7.15, neonatal intensive care unit (NICU) admission, duration of neonatal hospitalization, respiratory distress, need for phototherapy, intrauterine growth restriction, and nuchal cord.

### Statistical analysis

Continuous variables were expressed as mean ± standard deviation (SD) or median (range) and compared using Student’s t-test or the Mann–Whitney U test, as appropriate. Categorical variables were expressed as numbers (percentages) and compared using chi-square or Fisher’s exact test. A two-sided p-value < 0.05 was considered statistically significant.

## Results:

### Baseline characteristics

A total of 413 primiparous patients presenting with DFM at 39 weeks were included: 282 in the induction group and 131 managed expectantly. Maternal age, gravidity, previous miscarriages, medical comorbidities, ethnicity, residence, and GBS colonization were comparable between groups (Table 1). However, Bishop score at admission was significantly less favorable in the induction group, with a higher proportion of patients presenting with a score < 6 (95.4% vs. 87.2%, p = 0.011). Rates of balloon catheter ripening (68.2% vs. 16.0%, p = 0.001), dinoprostone use (8.7% vs. 1.6%, p = 0.014), and oxytocin administration (78.9% vs. 44.8%, p = 0.001) were higher in the induction group. Overall rate of induction of labor in the expectant management group was 59/131 (45.0%).

**Table 1:** Baseline maternal and obstetric characteristics of primiparous patients presenting with DFM at 39 weeks, by management strategy

Variable	Induction Group (n = 282)	Control Group (n = 131)	p-value
Maternal age	26.9 ± 4.4	26.6 ± 4.0	0.469
Gravidity	1.2 ± 0.6	1.2 ± 0.6	0.763
Previous miscarriages	51 (18.1%)	26 (20.3%)	0.69
Gestational diabetes	1 (0.4%)	0 (0.0%)	0.818
Chronic hypertension	8 (2.8%)	1 (0.8%)	0.224
Full prenatal care	256 (91.1%)	115 (89.8%)	0.823
Ethnicity: Arab	177 (63.0%)	70 (54.7%)	0.21
Ethnicity: Jewish	101 (35.9%)	55 (43.0%)	0.21
Residence: City	113 (40.4%)	47 (37.3%)	0.406
Residence: Rural	164 (58.6%)	79 (62.7%)	0.406
GBS colonization	47 (16.7%)	13 (9.8%)	0.782
Bishop score at admission < 6	269 (95.4%)	116 (87.2%)	0.011
Bishop score 6–8	12 (4.3%)	16 (12.0%)	0.011
Bishop score > 8	1 (0.4%)	1 (0.8%)	0.011
Dinoprostone cervical ripening	24 (8.7%)	2 (1.6%)	0.014
Balloon catheter cervical ripening	191 (68.2%)	20 (16.0%)	0.001
Oxytocin administration	221 (78.9%)	56 (44.8%)	0.001
First complaint for DFM	207 (73.4%)	101 (78.9%)	0.284
Week of DFM presentation	39.3 ± 0.3	39.3 ± 0.3	0.17

DFM- decreased fetal movement

Data are presented as mean ± standard deviation (SD) or number (percentage), as appropriate

### Maternal outcomes

Maternal outcomes are shown in Table 2. Patients in the induction group delivered earlier (39.4 ± 0.3 vs. 40.1 ± 0.3 weeks, p < 0.001) and had a shorter interval from first DFM complaint to delivery (1.6 ± 0.3 vs. 3.2 ± 1.4 days, p < 0.001). Rates of intrapartum fever (12.8% vs. 10.5%, p = 0.917), clinical chorioamnionitis (7.4% vs. 3.8%, p = 0.219), and mode of delivery (vaginal delivery 72.3% vs. 75.9%; cesarean 20.9% vs. 17.3%; vacuum 6.7% vs. 6.8%) did not differ significantly between groups. Indications for cesarean (non-reassuring fetal heart rate vs. lack of progress) were also similar. Total hospitalization duration was longer in the induction group (3.6 ± 0.6 vs. 2.2 ± 0.5 days, p < 0.001), though postpartum stay was slightly shorter (2.0 ± 0.2 vs. 2.2 ± 0.1 days, p < 0.001).

**Table 2:** Maternal outcomes in primiparous patients presenting with DFM at 39 weeks: induction versus expectant management

Variable	Induction Group N = 282	Control Group N = 131	p-value
Recurrent complaint for DFM	75 (26.6%)	30 (22.1%)	0.284
Days from DFM to delivery	1.6 ± 0.3	3.2 ± 1.4	< 0.001
Delivery week	39.4 ± 0.3	40.1 ± 0.3	< 0.001
Duration of rupture of membranes (decimal hours)	6.8 ± 5.5	6.5 ± 5.5	0.605
Intrapartum fever	36 (12.8%)	14 (10.5%)	0.917
Chorioamnionitis	21 (7.4%)	5 (3.8%)	0.219
Duration of Second stage (decimal hours)	1.8 ± 1.2	1.8 ± 1.2	0.986
Vaginal delivery	204 (72.3%)	101 (75.9%)	0.683
Cesarean Delivery	59 (20.9%)	23 (17.3%)	0.683
Vacuum extraction	19 (6.7%)	9 (6.8%)	0.683
Cesarean due to non-reassuring fetal heart rate	32 (11.3%)	13 (9.8%)	0.755
Cesarean due to lack of progress	22 (7.8%)	5 (3.8%)	0.139
Total Hospitalization Duration (from triage admission through induction to postpartum discharge)	3.6 ± 0.6	2.2 ± 0.5	< 0.001

DFM- decreased fetal movement

Data are presented as mean ± standard deviation (SD) or number (percentage), as appropriate

### Neonatal outcomes

Neonatal outcomes are shown in Table 3. Neonatal outcomes were largely similar between the induction and expectant management groups. The proportion of neonates with cord pH < 7.15 was lower in the induction group than in the expectant management group (2.5% vs. 6.9%, p = 0.034). No cases of 5-min Apgar score < 7 were observed in either group. The mean duration of neonatal hospitalization was shorter in the induction group (2.0 ± 0.2 vs. 2.3 ± 0.5 days). The rates of nuchal cord, intrauterine growth restriction, NICU admission, respiratory distress, and need for phototherapy were comparable between groups.

**Table 3:** Neonatal outcomes in primiparous patients presenting with DFM at 39 weeks: induction versus expectant management

Variable	Induction Group N = 282	Control Group N = 131	p-value
Nuchal cord	18 (6.4%)	13 (9.9%)	0.220
Birthweight (g)	3326.3 ± 394.1	3323.3 ± 417.2	0.945
Intrauterine growth restriction	20 (7.1%)	12 (9.2%)	0.492
NICU admission	9 (3.2%)	6 (4.5%)	0.696
Neonatal hospitalization length (days)	2.0 ± 0.2	2.3 ± 0.5	< 0.001
Apgar 5 min < 7	0 (0.0%)	0 (0.0%)	0.921
Respiratory distress	6 (2.1%)	1 (0.8%)	0.543
Cord pH < 7.15	7 (2.5%)	9 (6.9%)	0.034
Need for phototherapy	33 (11.7%)	16 (12.0%)	0.786

DFM- decreased fetal movement, NICU neonatal intensive care unit

Data are presented as mean ± standard deviation (SD) or number (percentage), as appropriate

## Discussion

### Main findings

Among 413 primiparous patients presenting with DFM at 39 weeks, baseline characteristics were similar between groups, though cervical status was less favorable in those induced. Induction was associated with earlier delivery and shorter time to birth, without differences in cesarean rates or maternal complications. Neonatal outcomes were largely comparable; however, the induction group showed fewer cases with pH < 7.15, while other measures—including birthweight, Apgar scores, NICU admissions, and hospitalization duration—did not differ significantly.

### Interpretation

Our findings should be interpreted in the context of existing literature. Previous studies have consistently demonstrated that DFM in late pregnancy is linked to an increased risk of adverse outcomes—including stillbirth, low Apgar scores, NICU admission, and small-for-gestational-age infants—as largely reflective of placental insufficiency [7, 10]. On this basis, several guidelines, including the RCOG Green-top Guideline, recommend considering induction at term when patients report DFM in the absence of reassuring findings [12]. Consistent with this, we found that induction was associated with expedited delivery without excess maternal or neonatal morbidity. Although fewer neonates in the induction group had cord pH < 7.15, the clinical significance of this finding is uncertain, as it was not accompanied by improvements in clinically relevant neonatal outcomes such as NICU admission. Nearly half of the expectantly managed patients eventually underwent induction, reflecting ongoing clinical concern despite initial reassurance. These findings should be interpreted in the context of international variation in 39-week induction policies following the ARRIVE trial [19, 20]. While some U.S. institutions now routinely offer elective induction at 39 weeks to low-risk nulliparas, this practice is far from universal. Many countries, including Israel, Australia and much of Europe [21, 22], continue to use selective rather than routine induction, and in these settings the clinical scenario we examined remains common. Even in ARRIVE-implementing systems, patients may still present with DFM before their scheduled induction or when elective induction is not pursued due to patient preference or resource constraints. In such cases, our results suggest that immediate induction is a safe option that may confer neonatal acid–base advantages without increasing operative delivery or morbidity, supporting the relevance of these findings across diverse clinical environments. Interestingly, Akselsson et al. [23] reported that patients presenting with decreased or altered fetal movements who were managed conservatively after a reassuring evaluation frequently underwent subsequent induction of labor, with the likelihood of induction increasing in proportion to the number of presentations. Turner et al. [10] conducted a large population-based cohort study including 101,597 singleton pregnancies after 28 weeks’ gestation, of which 8,821 (8.7%) presented with DFM. Their study compared pregnancy outcomes between patients who presented with DFM and those who did not, rather than evaluating induction versus expectant management among those with DFM. Although presentation with DFM was associated with higher rates of obstetric intervention—including induction of labor, emergency cesarean delivery, and planned early-term birth—it was not associated with increased rates of stillbirth or significant perinatal morbidity. Importantly, their analysis encompassed all gestational ages beyond 28 weeks and did not specifically examine term or late-term pregnancies (39–39 + 6 weeks), which limits direct comparison to our study focusing on management decisions at term. In contrast, Majumdar et al. [9] conducted a prospective observational study including 83 pregnancies between 34 and 40 weeks complicated by DFM and reported elevated rates of adverse perinatal outcomes, likely due to the inclusion of patients with additional risk factors such as abnormal fetal heart rate tracings or fetal growth restriction. These findings highlight the heterogeneity of DFM populations and suggest that the prognostic significance of DFM depends largely on baseline risk and the presence of coexisting abnormalities.

Parity itself deserves consideration. Previous studies have shown that nulliparous patients are overrepresented among those presenting with DFM. McCarthy et al. [24] found that half of patients seeking care for DFM were nulliparous compared with only one-third in the reference population, and a Norwegian study reported similar results (51% vs. 39%) [25]. Nulliparity may thus be a predisposing factor for heightened concern or reporting of DFM, underscoring the relevance of restricting our cohort to primiparas.

Patterns of management may vary by country; for example, in Ireland, 42% of patients presenting with DFM beyond 28 weeks underwent induction of labor compared with 28% in the general obstetric population [24], illustrating how clinical practice patterns can influence intervention rates independent of perinatal risk. In our study, all primiparous patients presenting at 39 weeks with isolated DFM were uniformly offered induction of labor according to departmental protocol. Notably, 68.3% of these patients accepted induction, while the remainder opted for expectant management, enabling a direct comparison of outcomes between the two approaches. Subsequent studies [17, 26, 27] have explored similar interventions designed to enhance awareness of fetal activity, such as structured fetal movement counting, kick charts, daily tracking applications, and educational programs. Hayes et al. [26] showed that these approaches may reduce NICU admissions and low Apgar scores without increasing induction or cesarean rates, while Akselsson et al. [27] demonstrated that the “Mindfetalness” program was associated with lower rates of induction, cesarean delivery, and small-for-gestational-age outcomes. Unlike these population-based or behavioral interventions, our study focused on the management of primiparous patients with isolated DFM at 39 weeks and reassuring fetal assessment, which may explain the favorable neonatal outcomes observed across both management strategies.

Taken together, our findings contribute to the ongoing debate regarding the optimal management of patients presenting with DFM beyond 39 weeks. The observation that induction expedited delivery without worsening maternal or neonatal outcomes supports its safety in this context. Equally, the absence of excess morbidity in the expectant management group suggests that careful surveillance may also be appropriate for selected patients. This highlights the importance of individualized counseling that incorporates cervical status, maternal preferences, and institutional resources. In settings where both options are feasible, offering patients the choice between induction and expectant management may be reasonable, provided that fetal status is reassuring, and close fetal monitoring is ensured. Refinement of risk stratification tools is needed to identify which patients benefit most from immediate intervention and which can safely await spontaneous labor.

### Strengths and limitations

The strengths of our study include the relatively large and well-defined cohort, strict inclusion and exclusion criteria, and standardized induction protocols, all of which enhance internal validity. Nonetheless, several limitations should be acknowledged. The retrospective design carries inherent risk of residual confounding and prevents causal inference. The single-center setting may limit generalizability to other populations and health systems. Patient self-selection into induction or expectant management introduces potential bias, as maternal preferences and subtle clinical factors may have influenced decisions. Long-term neonatal outcomes were not available, precluding assessment beyond the immediate postpartum period. Our findings apply specifically to primiparous patients, as multiparas were excluded to maintain a homogeneous cohort, and exclusion of high-risk pregnancies further limits generalizability to low-risk populations. Finally, although adequately powered for common maternal and neonatal outcomes, the study was underpowered to evaluate rare but critical events such as stillbirth. Despite these limitations, the study provides important real-world evidence regarding management of isolated DFM at 39 weeks.

## Conclusion

Our study suggests that among primiparous patients presenting at 39 weeks with isolated DFM and reassuring fetal assessment, both induction of labor and expectant management are associated with favorable maternal and neonatal outcomes. Induction provides the advantage of earlier delivery without increasing operative delivery rates or neonatal morbidity, whereas expectant management remains a safe alternative when accompanied by close surveillance. These findings emphasize the importance of individualized counseling and shared decision-making, taking into consideration cervical status, maternal preferences, and institutional protocols. Future prospective multicenter studies are warranted to validate these results, evaluate long-term outcomes, and refine risk stratification strategies to optimize management for patients presenting with DFM.

## Data Availability

Yes. This study used clinical data generated as part of the research. The dataset analyzed during the current study is not publicly available due to patient confidentiality and institutional policy but is available from the corresponding author on reasonable request.
